# Supplementary material for: Systematic Identification and Functional Analysis of the Hypericum perforatum L. bZIP Gene Family Indicating That Overexpressed HpbZIP69 Enhances Drought Resistance
Source: Int J Mol Sci. 2023 Sep 18;24(18):14238. doi: 10.3390/ijms241814238 (PMC10531856; doi:10.3390/ijms241814238)
Supplement: Supplementary file 1 [file ijms-24-14238-s001.zip › ijms-2536327-supplementary figures.pdf]

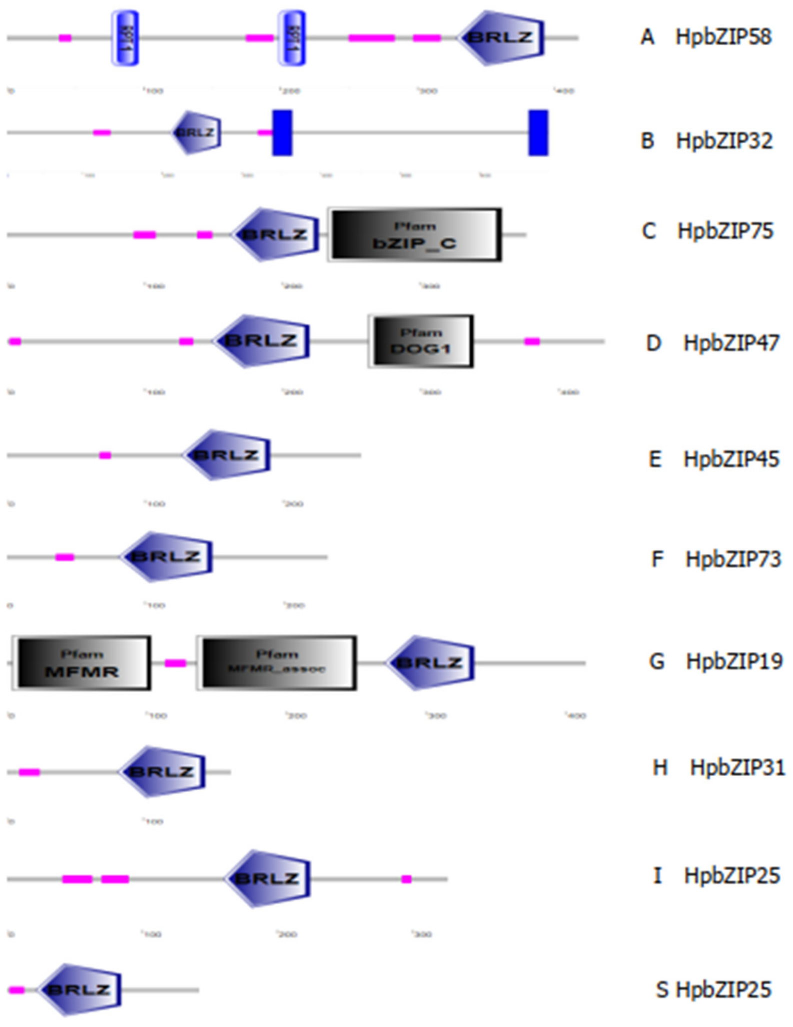

Figure S1: Structure of representative HpbZIP proteins from each subgroup.

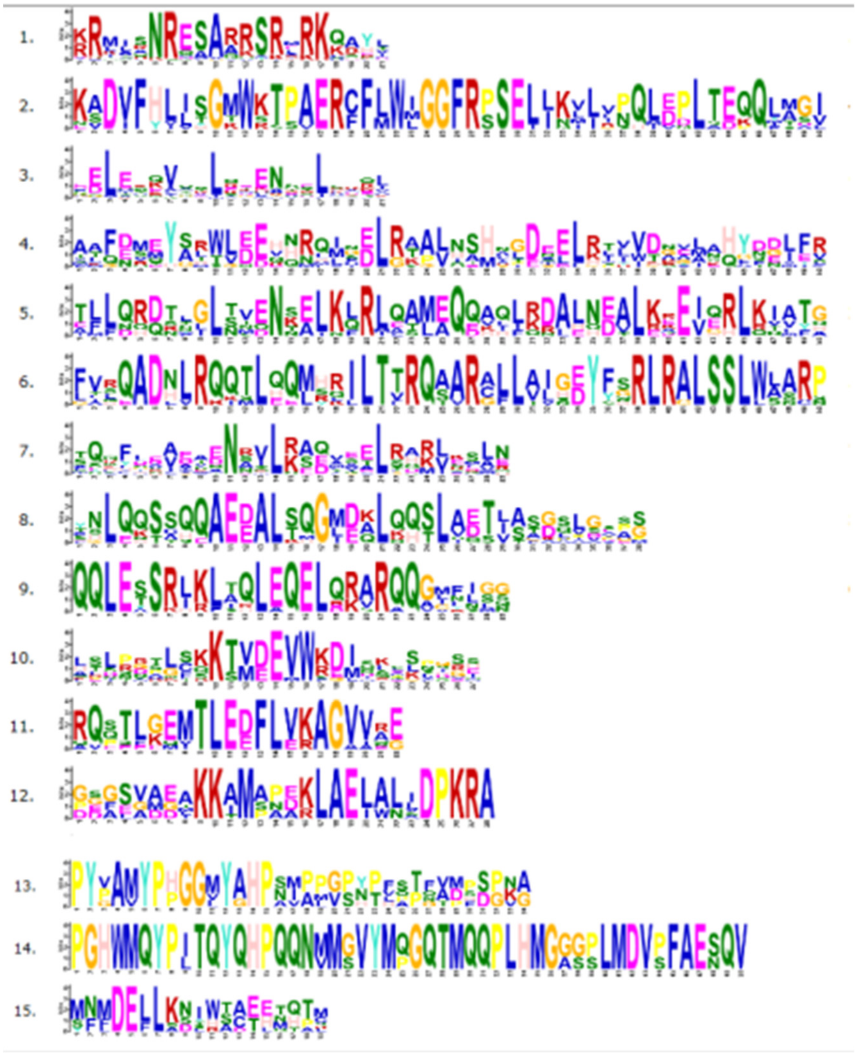

Figure S2: Seqlogo of conserved motifs in HpbZIP proteins.

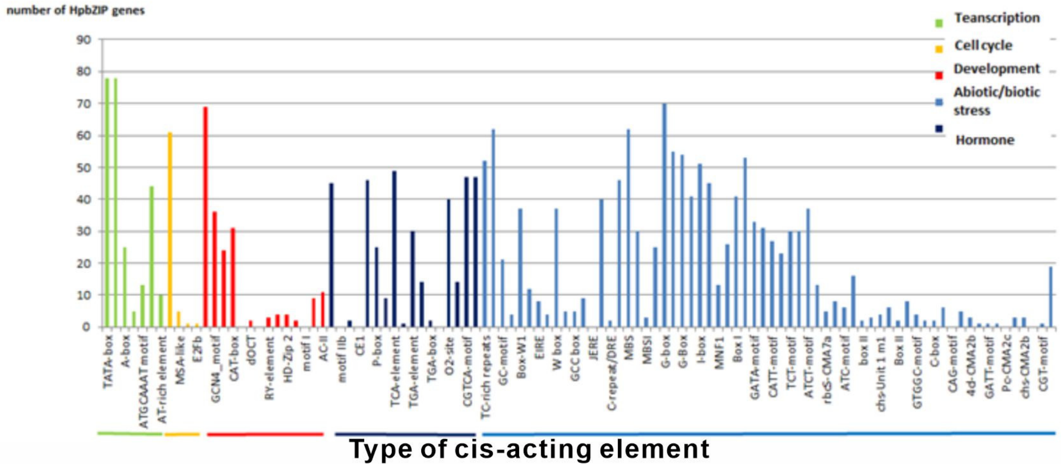

Figure S3: Cis-Acting elements analysis of *HpbZIP* gene promoters. The cis-acting elements were identified by the online PlantCARE program using 1.5 kb upstream of transcription initiation site of *HpbZIP* genes. The graph was generated based on the presence of cis-acting elements in response to specific processes /elicitors/conditions (x-axis) in *HpbZIP* family members (y-axis).

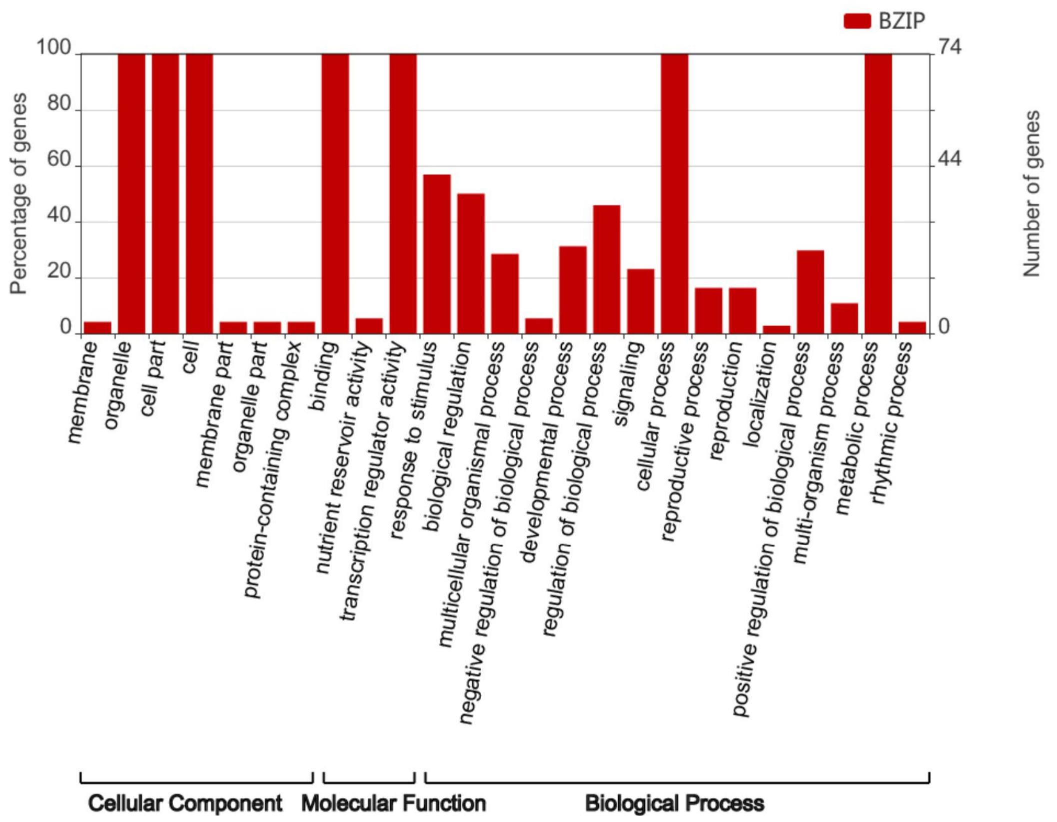

Figure S4: Gene Ontology annotation results for HpbZIP proteins. The y-axis on the left side indicates the percentage of a specific category of genes in the main category, and the right side indicates the number of genes in a category.

14 Nearest Neighbors

id	site	distance	identity	comments
HSF8_LYCPE	nucl	213.887	12.7135%	[Uniprot] SWISS-PROT45:Nuclear.
AG_BRANA	nucl	227.742	12.5668%	[Uniprot] SWISS-PROT45:Nuclear.
AGL1_ARATH	nucl	239.361	12.5668%	[Uniprot] SWISS-PROT45:Nuclear.
GBF1_ARATH	nucl	243.374	13.1016%	[Uniprot] SWISS-PROT45:Nuclear.
GBF2_ARATH	nucl	246.724	11.7021%	[Arath] [Uniprot] SWISS-PROT45:Nuclear. Evidence:IDA Pubmed:9193069
GBF3_ARATH	nucl	252.626	10.1828%	[Uniprot] SWISS-PROT45:Nuclear.
CPR3_PETCR	nucl	254.56	13.9037%	[Uniprot] SWISS-PROT45:Nuclear.
At2g34900.1	nucl	255.847	10.5398%	[Arath]
HFR1_ARATH	nucl	255.915	10.1604%	[Uniprot] SWISS-PROT45:Nuclear.
At5g54110.1	plas	257.636	11.2299%	[Arath]
HAT5_ARATH	nucl	260.988	11.2299%	[Uniprot] SWISS-PROT45:Nuclear.
HKN1_MAIZE	nucl	262.871	11.2299%	[Uniprot] SWISS-PROT45:Nuclear.
At1g33760.1	nucl	262.93	11.4973%	[Arath]
HSF8_LYCES	nucl	264.341	12.9032%	[Uniprot] SWISS-PROT45:Nuclear.

HpbZIP69

Figure S5: Predicted subcellular localization of HpbZIP69 using WoLFPSOR (https://wolfpsort.hgc.jp/).
